# The Impact of Early Specialist Management on Outcomes of Patients with In-Hospital Stroke

**DOI:** 10.1371/journal.pone.0104758

**Published:** 2014-08-21

**Authors:** Dulka Manawadu, Jithesh Choyi, Lalit Kalra

**Affiliations:** 1 Department of Clinical Neuroscience, King’s College London, London, United Kingdom; 2 Acute and Emergency Medicine, King’s College Hospital, London, United Kingdom; Cardiff University, United Kingdom

## Abstract

Delays in treatment of in-hospital stroke (IHS) adversely affect patient outcomes. We hypothesised that early referral and specialist management of IHS patients will improve outcomes at 90 days. Baseline characteristics, assessment delays, thrombolysis eligibility, 90-day functional outcomes and all-cause mortality were compared between IHS patients referred for specialist stroke management within 3 hours of symptom onset (early referrals) and later referrals. Patients were identified from a prospective stroke registry between January 2009 and December 2010. Inclusion criteria were primary admission with a non-stroke diagnosis, onset of new neurological deficits after admission and early ischaemic changes on CT or MR imaging. Eighty four (4.6%) of 1836 stroke patients had IHS (mean age 74 year; 51% male, median NIHSS score 10). There were no significant differences in baseline characteristics between 53 (63%) early and 31 (37%) late referrals. Thrombolysis was performed in 29 (76%) of the 37/78 (47%) potentially eligible patients; 7 patients were excluded because specialist referral was delayed beyond 4.5 hours despite symptom recognition within 3 hours of onset. Early referral improved functional outcomes (modified Rankin Scale 0–2 at 90 days 40% v 7%, p = 0.001) and was an independent predictor of mRS 0–2 at 90 days after adjusting for age, pre-morbid function, primary cause for hospital admission and stroke severity [OR 1.13 (95% C.I.  = 1.10–1.27), p = 0.002]. Early referral and specialist management of IHS patients that includes thrombolysis is associated with better functional outcomes at 90 days.

## Introduction

Nearly 4–15% of all strokes occur in hospitalised patients, [1]–[3] and are associated with higher in-hospital mortality and poorer functional status at discharge compared with out of hospital stroke patients. [1], [4] Although additional co-morbidities, metabolic or haematological derangements and greater stroke severity in IHS patients are major contributors to poor outcomes, [5] many studies have suggested that delays in recognition, referral, specialist assessment and thrombolysis may also play an important role. [6], [7] Findings suggest that only 25%–50% of in-hospital stroke (IHS) patients are assessed within 3 hours of symptom onset despite already being in a hospital, [6], [7] and 15% of IHS patients may be denied thrombolysis because of these delays. [6] This is important as studies show that the safety and 90 day outcomes of thrombolysis of IHS patients are similar to those who present from outside hospital to emergency departments [8].

It has been suggested that early assessment for thrombolysis and specialist stroke care may improve outcomes in IHS patients, [3], [6]–[8] but there is little direct evidence to support this assumption. Even if IHS patients are not eligible for thrombolysis, early access to stroke-specific specialist care for optimisation of blood pressure and other physiological parameters, prevention of stroke related complications and treating for stroke extension or recurrence may improve mortality and functional outcomes in these patients. [9] We hypothesised that early referral and specialist management of IHS patients will be associated with lower mortality and better functional outcomes at 90 days.

The main objective of the study was to compare patient profiles, stroke characteristics and 90 day outcomes for in-hospital stroke patients referred to and managed by the specialist stroke team within 3 hours of stroke onset compared with those in whom referral and specialist management was delayed. We also estimated the frequency of in-hospital stroke, eligibility for thrombolysis and the effect of thrombolysis on outcomes.

## Methods

Data were extracted from a prospective register of all consecutive stroke patients presenting to an academic hospital between January 2009 and December 2010. The hospital has a full range of emergency, secondary and tertiary specialities and serves a local population of 500,000 in South London. In-hospital stroke was defined as: 1) primary admission to hospital with a non-stroke diagnosis; 2) clear onset of new neurological deficits after admission fulfilling the clinical definition of a stroke; 3) Early ischaemic changes [10] and/or hyperdense artery sign [11] on CT or DWI lesions on MR imaging. In addition to the register, inadvertently missed cases were identified from multiple overlapping sources including hospital patient databases using ICD-9 codes for the stroke cluster of diagnoses, imaging requests and death certificates. The case notes for these patients were reviewed by at least 2 investigators to confirm new diagnosis of stroke and 6 additional IHS patients were added to the database. Data extracted from the registry were verified against source data for completeness and accuracy. In keeping with NHS Policy, ethical approval for interrogation of the clinical database for this study was approved by the East Midlands National Research Ethics Service Committee (11/EM/0190) on behalf of the institution. Written informed consent for use of clinical records was not obtained from patients or next of kin but patient records were anonymised prior to analysis.

Specialist assessment and management was undertaken by stroke fellows and included National Institute of Health Stroke Scale (NIHSS) score, review of imaging, thrombolysis if appropriate, initiation of vascular investigations and anti-thrombotic treatment as appropriate, and transfer to stroke unit for physiological optimisation, prevention of complications and specialist multidisciplinary management. Data were collected on vascular risk profile, pre-morbid functional status, primary cause for hospital admission, in-patient location, comorbidities, stroke aetiology, blood pressure, blood glucose, and duration between hospital admission and stroke onset, stroke onset and recognition of symptoms, specialist assessment, imaging and thrombolysis or other treatments.

The primary outcome was the dichotomised mRS, defined as excellent (mRS 0–1) or favourable (mRS 0–2), and assessed at 90 days during a clinic visit or telephone by certified assessors not involved in patient care. New symptomatic intracerebral haemorrhage (sICH) in thrombolysed patients defined by the European Cooperative Acute Stroke Study (ECASS-II) classification. [12] Other outcomes were all-cause and stroke-related mortality at 90 days (sICH, cerebral oedema, stroke extension or stroke related complications such as aspiration pneumonia or pulmonary embolism).

### Statistical Analysis

Baseline characteristics, processes of care and 90 day outcomes were compared between IHS patients referred to and managed by the specialist stroke team within 3 hours of symptom onset (early referrals) and those referred and assessed later (late referrals). All analyses were pre-specified prior to data extraction. Data were tested using the Kolmogorov-Smirnov test and are presented as means (standard deviation) or medians (interquartile range). Comparisons were undertaken using Chi-square test, Fisher exact test, independent samples t-test or Mann Whitney test as appropriate. The association between baseline prognostic characteristics of age, sex, vascular risk factor profile, blood pressure, blood glucose, stroke severity and aetiology, delay in referral and specialist assessment and thrombolysis with the outcomes of mRS 0–2, mortality and sICH were assessed using Pearson’s and Spearman’s correlation tests as appropriate. In addition, this analysis also included history of myocardial infarction or congestive heart failure, pre-morbid mRS score and index reason for hospital admission (dichotomised as those for TIA, elective cardiac or other investigations or procedures and non-elective or major surgical/medical interventions) to adjust for patient characteristics and associated conditions being responsible for delay in referral and discrepancies in outcome. Variables found to be significant at p<0.10 level in these analyses were entered into conditional logistic regression models using stepwise backward deletion to evaluate the independent effect of early referral and specialist management on outcome. The model fit was assessed using the Hosmer-Lemeshow test and models with p<0.10 were discarded. The predictive value of the model was assessed by Cox and Snell’s R^2^ test. Two-sided p values of <0.05 were considered significant. IBM-SPSS version 20.0 was used for analyses.

## Results

**Figure 1:**
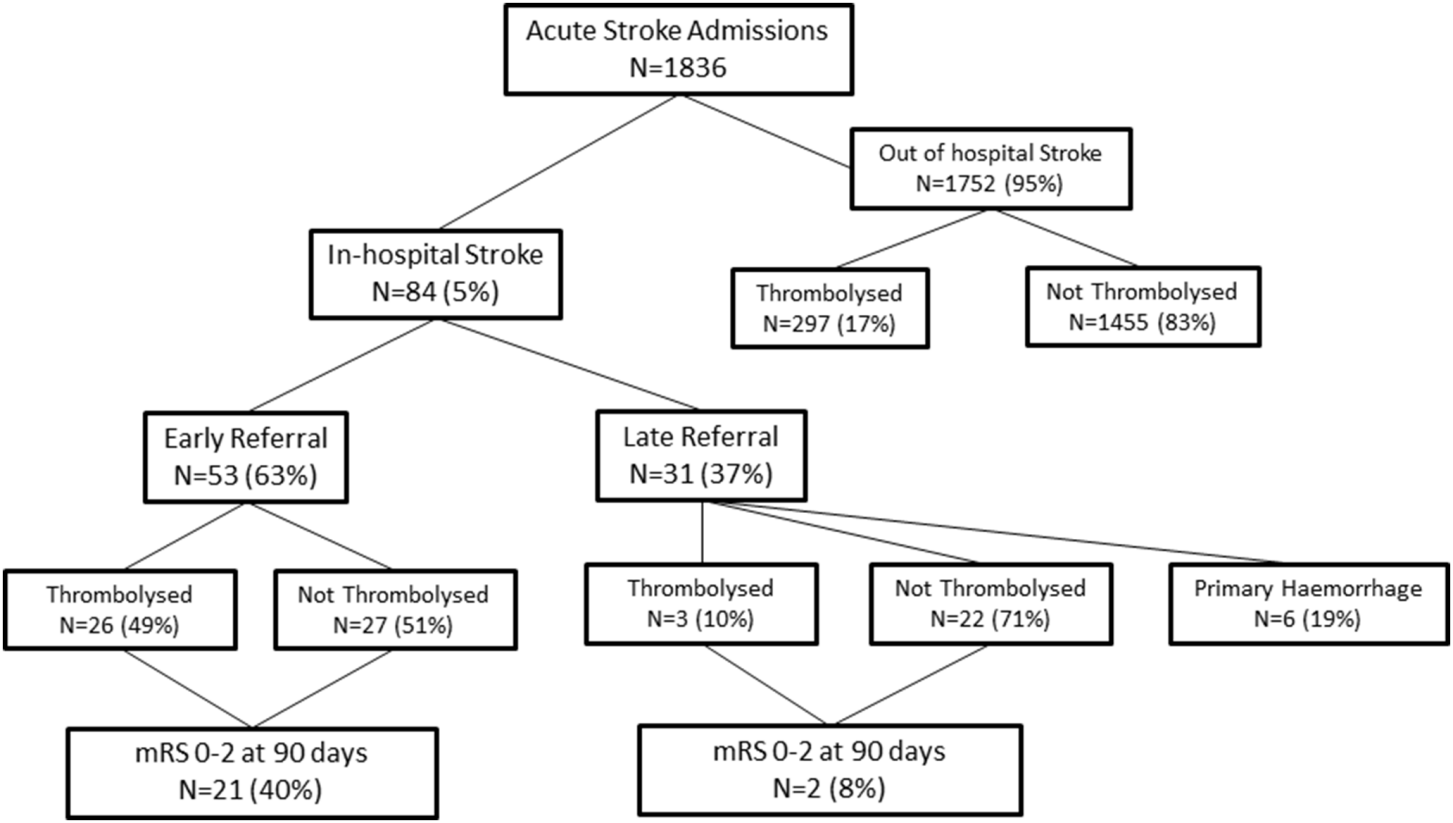
Flow of patients included in the study between Jan 2009 to December 2010.

There were 1836 acute stroke admissions during the 2 year duration of the study, of which 84 (4.6%) were in-hospital strokes (Fig. 1). The reasons for the primary admission for IHS patients were: cardiac surgery or percutaneous intracardiac procedures = 15, myocardial infarction or heart failure = 13, cardiac angiography or coronary artery stenting = 6, transient ischaemic attack for investigation = 9, neurosurgical procedures = 2, orthopaedic, abdominal or vascular surgery = 12, carcinomatosis = 6, internal medicine admissions including sepsis, pneumonia, diabetic ketoacidosis and other metabolic disorders, hepatic, respiratory or renal failure = 21. The median duration from primary hospital admission to stroke onset was 2 days (IQR 1–6 days). Ischaemic strokes were seen in 78 (93%) IHS patients and haemorrhagic stroke in 6 patients. Thrombolysis was undertaken in 29 IHS patients with ischaemic strokes and accounted for 9% of the 326 patients thrombolysed during the study period.

The baseline characteristics of IHS patients are given in Table 1. Of the 84 IHS patients, 67 (80%) were recognised within 3 hours of symptom onset and, of these, 53 (79%) were early referrals. Reasons for delayed referral despite early recognition included need for cardiac monitoring (n = 4), misdiagnosis as an acute confusional state (n = 3) and CT imaging showing intracranial haemorrhage (n = 6). The time of onset was not known in 17/84 (20%) patients despite being in a hospital. There were no significant differences in the age, sex, vascular risk profile or baseline stroke severity between early and late referrals (Table 1). Cardiac disease and procedures were the commonest primary diagnosis and cardio-embolism was the predominant stroke aetiology in both groups.

**Table 1:** Baseline patient and stroke characteristics of 84 in-hospital stroke patients.

	Early Referrals n = 53	Late referrals N = 31	P value
**Patient characteristics**			
Mean (SD) age in years	75.1 (13.1)	73.4 (12.1)	0.57
Male (%)	27 (51)	16 (52)	0.95
Hypertension (%)	43 (81)	29 (94)	0.12
Diabetes (%)	18 (34)	10 (32)	0.87
Dyslipidaemia (%)	16 (30)	9 (29)	0.12
Atrial fibrillation (%)	22 (42)	12 (39)	0.80
Current smoker	16 (30)	7 (23)	0.54
Anti-platelets or anticoagulants	37 (70)	21 (68)	0.88
Pre-admission mRS 0–2	41 (77)	18 (58)	0.062
**Reasons for primary admission (%)**			0.001
TIA	9 (100)	0	
Percutaneous cardiac intervention	12 (100)	0	
Post MI/CHF	5 (71)	2 (29)	
Post-cardiac surgery	11 (58)	8 (42)	
Internal medical admissions	11 (46)	13 (54)	
General and orthopaedic surgery	5 (38)	8 (62)	
Med. days in hospital pre-stroke (IQR)	3 (1–6)	2 (1–8)	0.49
**Baseline assessments**			
Mean (SD) SBP (mm Hg)	136 (33)	132 (39)	0.67
Mean (SD) DBP (mm Hg)	75 (18)	76 (17)	0.89
Mean (SD) glucose (mmol/l)	8.8 (5.9)	8.8 (3.4)	0.99
Median (IQR) NIHSS score	10 (7;16)	10 (9;16)	0.59
**Stroke Aetiology**			0.14
Atherosclerotic	7 (13)	6 (19)	
Cardioembolic	36 (68)	17 (55)	
Lacunar	6 (11)	2 (7)	
Mixed aetiology	4 (8)	0	
Intracranial Haemorrhage	0	6 (20)	

Early referral of IHS patients to stroke specialist teams was associated with significantly shorter specialist assessment, imaging and treatment times compared with late referrals (Table 2). A significantly greater proportion of early referral patients had appropriate vascular investigations undertaken within 24 hours and transferred to specialist stroke care. Antithrombotic treatment was reviewed and antiplatelet or anticoagulant treatment initiated much earlier if appropriate in these patients (Table 2).

**Table 2:** Comparison of processes of care between early and late referrals of in-hospital stroke patients.

	Thrombolysed N = 29	Early referrals N = 53	Late referrals N = 31	P- value (early v late)
**Processes of care**				
Onset to recognition	00∶00	00∶00	01∶50*	0.002
Recognition to referral	00∶14	00∶26	16∶00	0.0001
Recognition to CT imaging†	00∶22	00∶59	01∶44	0.61
Referral to specialist assessment	00∶24	00∶37	15∶58	0.0001
Scan to thrombolysis	00∶24	-	-	
Onset to thrombolysis	01∶20	-	-	
Onset to ATT review/initiation	-	02∶06	36∶54	0.0001
Specialist stroke unit in 4 h	-	44 (83%)	9 (29%)	0.009
Vascular Imaging in 24 h	-	53 (100%)	12 (39%)‡	0.011
Echocardiography in 24 h	-	28 (53%)	13 (42%)	0.57

All times are medians and expressed as (hh∶mm).

*excludes patients with unknown time of onset.

† CT scan may be undertaken prior to specialist referral.

‡ significant at p<0.02 even when the 6 ICH patients are excluded.

In-hospital stroke patients who were referred early for specialist management showed greater early improvements and better functional outcomes at 90 days (Table 3). This difference was present even when the 6 patients with haemorrhagic stroke, who were referred late and expected to have a worse outcome, were excluded from the analyses. There were no differences in end of life care decisions or all-cause mortality, which in most patients were due to non-stroke related causes. Early referral for specialist management was an independent predictor of mRS 0–2 at 90 days after adjusting for age, pre-morbid function, primary cause for hospital admission, NIHSS score and thrombolysis in ischaemic IHS stroke patients (Table 4). This independent effect of early referral and specialist management was also true for the whole group (n = 84) that included haemorrhagic stroke patients [OR 1.13 (95% C.I.  = 1.10–1.27), p = 0.002]. Other independent predictors of good outcome were younger age, primary admission for TIA or percutaneous cardiac procedures and lower baseline NIHSS score. Diabetes and a pre-morbid mRS score >2 independently predicted poor outcomes and higher mortality at 90 days (Table 4).

**Table 3:** Comparison of outcomes between early and late referrals of in-hospital stroke patients.

	ThrombolysedN = 29	EarlyreferralsN = 53	LatereferralsN = 31	Late referrals(excl ICH)N = 25	P- value(early v late)
**Outcomes**					
Median (IQR) NIHSS at 24 hours	8 (2∶16.5)	8 (4; 11)	9 (7∶14)	9 (7.5; 14)	0.09
Median (IQR) NIHSS change	−3 (−11;0)	−1 (−4; 0)	0 (−2,2)	0 (−2; 3)	0.014
mRS 0–1 at 90 days (%)	4 (14)	9 (17)	1 (3)	1 (4)	0.60
mRS 0–2 at 90 days (%)	10 (34.5)	21 (40)	2 (7)	2 (8)	0.001
Any new ICH at 24 hours (%)	8 (27%)	8 (15)	1 (3)	1 (4)	0.040
New sICH at 24 h (%)	2 (7)	2 (4%)	0	0	0.16
End of life care decision	-	5 (9)	6 (19)	4 (13)	0.42
90 day all-cause mortality (%)	12 (41)	16 (32)	11 (37)	9 (36)	0.74
90 day stroke-related mortality (%)	4 (14)	4 (8)	7 (23)	5 (20)	0.11

Baseline NIHSS score of thromblysed patients was 14 compared to 10 in other groups.

**Table 4:** Predictors of good functional outcome (mRS 0–2) and morality at 90 days and symptomatic intracranial haemorrhage (sICH) at 24 hours in IHS patients with an ischaemic stroke (n = 78).

Outcome	Determinants	Univariate correlation		Logistic regression	
		Coefficient	P	OR (95% C.I.)	p
mRS 0–2 at 90 days	Age	−0.413	0.0001	0.92 (0.85–0.99)†	0.048
	Index diagnosis*	0.53	0.0001	1.41 (1.20–1.80)	0.011
	Diabetes	−0.338	0.002	0.06 (1.01–1.44)	0.006
	Pre-morbid mRS>2	−0.457	0.0001		
	Baseline NIHSS score	−0.387	0.001	0.83 (0.71–0.97)†	0.023
	Early specialist management	0.324	0.004	1.04 (1.01–1.46)	0.007
	Thrombolysis	0.084	0.46		
sICH at 24 h	No predictors				
	Thrombolysis	0.176	0.123		
All-cause mortality at 90 days	Index diagnosis*	−0.415	0.0001	0.39 (0.07–0.82)	0.014
	Diabetes	0.235	0.043		
	Pre-morbid mRS>2	0.609	0.0001	1.17 (1.04–1.67)	0.011
	Baseline NIHSS score	0.245	0.034		
	Early specialist management	−0.324	0.004		
	Thrombolysis	−0.112			

*Index diagnosis of TIA, percutaneous cardiac procedures or acute myocardial infarction compared with surgical procedures and multiple medical co-morbidities.

† ORs for per year increase in age and per unit increase in NIHSS score.

R^2^ for mRS 0–2 = 0.52 and R^2^ for all-cause mortality = 0.24.

Eligibility criteria for thrombolysis (excluding duration since stroke onset) were met in 37/78 (47%) in-hospital patients with ischaemic stroke, 27 of whom had been referred early (Table 5). Thrombolysis could not be undertaken in 7 (19%) potentially eligible but late referrals because specialist referral was delayed beyond 4.5 hours. One early referral patient on warfarin was not thrombolysed because of delays in obtaining INR results. Thrombolysis was undertaken in 29 (76%) patients (mean age 76.2±15.1 years, 52% male, median baseline NIHSS score 14 (IQR: 9.5; 17), 26 of whom received IV alteplase. Three patients with wake-up stroke in the late referral group underwent endovascular recanalization following multimodal CT imaging for perfusion mismatch and vessel occlusion. The decision to thrombolyse correlated with primary admission for TIA, percutaneous cardiac procedures or recent myocardial infarction (r = 0.45, p = 0.0001), early referral for specialist management (r = 0.53, p = 0.0001) and higher baseline NIHSS score (r = 0.36, p = 0.001). Thrombolysis was associated with significant early improvement despite two sICH at 24 hours but the 90 days outcomes were not different from patients referred for early specialist management (Table 3). Comparison between thrombolysed and non-thrombolysed patients in the early referral group was not possible because of non-comparability of eligibility for thrombolysis and small numbers. Thrombolysis was not independently associated with good functional outcomes or mortality at 90 days.

**Table 5:** Eligibility for thrombolysis in IHS patients with ischaemic stroke (n = 78).

	Early Referrals N = 53	Late referrals N = 25	P value
Thrombolysis eligibility*			0.37
Eligible (%)	27 (51%)	10 (40%)	
Non-eligible (%)	26 (49%)	15 (60%)	
Reasons for non-eligibility			
* Cardiac surgery/valve replacement*	*11*	*3*	
* Non-cardiac surgical procedure*	*5*	*4*	
* Advanced carcinomatosis*	*1*	*2*	
* Multiple medical co-morbidities*	*5*	*4*	
* Pre-morbid high dependence*	*4*	*2*	
Thrombolysed	26/27 (96%)	3/10 (30%)†	0.001

*excluding time criteria of stroke onset <4.5 hours.

† Three wake-up stroke patients thrombolysed “out of protocol” based on perfusion mismatch.

## Discussion

In-hospital strokes accounted for approximately 5% of all acute stroke admissions at a large multispecialty teaching hospital admitting all patients from a defined urban catchment. Although 80% of IHS patients were recognised within 3 hours of onset, only 60% were referred for specialist stroke management within 3 hours. The time of onset was not known in 20% patients despite being in a hospital and nearly 20% patients potentially eligible for thrombolysis were denied the treatment because of late referral. Early referral for specialist management was associated with earlier assessments, thrombolysis, specific vascular investigations and management on a stroke specialist unit, all of which contributed to better functional outcomes at 90 days.

Although thrombolysis is the prime driver for early referral of IHS to specialist services, [6], [8] only half of the IHS patients referred early were eligible for thrombolysis. In addition, this consecutive cohort of IHS patients showed poorer outcomes of thrombolysis than seen in a previous study limited to IHS patients selected for thrombolysis. [8] This difference in outcome can be attributed to the greater age and co-morbidity in our sample which was comparable in age, baseline characteristics, primary diagnoses, stroke aetiology and stroke severity to previous studies [3], [6] and representative of unselected hospital populations. The frequency of 4.6% for IHS in our sample also was comparable to 4.4% seen in a large registry of over 15,000 stroke patients, [1] suggesting a small target population for thrombolysis than seen in other studies. [2], [4] The overall benefits of thrombolysis in IHS and are likely to be influenced by the number of eligible patients, underlying co-morbidities and metabolic/haematological derangements in these patients.

A major limitation of the study is that it is a registry based non-randomised comparison of outcomes but it would be unethical to undertake a prospective study in which IHS patients were denied early specialist management. Sampling bias was minimised by pre-defining patient selection criteria in advance and including all consecutive stroke patients on to the register who met these criteria. Under-reporting bias was reduced by using multiple overlapping sources for ascertainment of IHS patients. Data were collected prospectively using standardised definitions and validated scales and verified against medical records for accuracy and completeness. Outcomes were assessed by trained observers not involved in patient care and all analyses were pre-specified. There were no differences in age, primary diagnoses, pre-morbid function, baseline NIHSS score or end of life care decisions that would favour good outcomes in early referral patients. Logistic regression adjusted for any differences in patient and stroke characteristics, baseline prognostic variables and reasons that may influence early referral such as pre-morbid function, co-morbidity and type or severity of initial medical presentation continued to show benefit with early referral and specialist management.

This is the first study to assess outcomes of IHS patients at 90 days and demonstrate that early referral of these patients for specialist management improves functional outcomes at 90 days. It is likely that several processes besides thrombolysis contribute to the beneficial effect of early specialist management of IHS patients and include faster assessments, imaging, appropriate thromboprophylaxis, blood pressure management and transfer to specialist stroke care, which has strong evidence for improving stroke outcomes. [9], [13] The study highlights the need for implementing processes that facilitate early recognition, referral and specialist management of in-hospital stroke patients.
